# Mixed-Pathogen Infections in Vegetatively Propagated Crops: From Biological Synergism to Integrated Management

**DOI:** 10.3390/plants15091332

**Published:** 2026-04-27

**Authors:** Juan M. Pardo, Nakarin Suwannarach, Srihunsa Malichan, Wilmer J. Cuellar, Wanwisa Siriwan

**Affiliations:** 1 Department of Plant Pathology, Faculty of Agriculture, Kasetsart University, Bangkok 10900, Thailand; j.m.pardo@cgiar.org (J.M.P.); srihunsa.m@ku.th (S.M.); 2 Crop Protection Research Area, Cassava Program Asia Office, International Center for Tropical Agriculture (CIAT), Vientiane P.O. Box 783, Laos; 3 Department of Biology, Faculty of Science, Chiang Mai University, Chiang Mai 50200, Thailand; suwan.462@gmail.com; 4 Crop Protection Research Area, Cassava Program, International Center for Tropical Agriculture (CIAT), The Americas Hub, Km 17 Recta Cali-Palmira, Cali 763537, Colombia

**Keywords:** vegetatively propagated crops, mixed-pathogen infection, synergism and seed degeneration

## Abstract

Vegetatively propagated crops, including cassava, sweet potato, banana, and potato, are susceptible to mixed-pathogen infections resulting from the continuous use of clonal planting material and infrequent seed replacement. A diverse array of viruses, bacteria, and fungi can accumulate within these materials over successive cultivation cycles, precipitating seed degeneration and complex disease syndromes that complicate diagnosis and management. Mixed infections frequently trigger synergistic interactions that exacerbate disease severity and yield losses. This review synthesizes data on mixed-pathogen complexes in vegetatively propagated crops, with particular focus on vascular and systemically colonizing pathogens and analyzing starch crops to highlight the epidemiological, biological, and ecological drivers of synergism and antagonism. Furthermore, the review examines host defense responses during coinfection, including the modulation of plant immune pathways, and evaluates how interpathogen dynamics influence pathological outcomes. Although advancements in molecular diagnostics—notably next-generation sequencing and metagenomics—have revolutionized the detection of mixed infections, they have also introduced challenges in differentiating causal agents from commensal microorganisms. Finally, we discuss the implications for integrated disease management, emphasizing clean seed systems, resistance breeding, and phenotyping strategies tailored to multipathogen environments. The dynamics of mixed infections is critical for resilient and sustainable management strategies amidst increasingly complex agricultural and climatic shifts.

## 1. Introduction

Mixed-pathogen infections in vegetatively propagated crops have emerged as a substantial concern in modern agriculture, fundamentally driven by the clonal nature of these systems. Clonal propagation, the asexual reproduction of plants, results in genetically identical offspring and is a method widely utilized for economically vital crops such as potato, banana, and sweet potato.

Clonal propagation ensures genetic uniformity, which is beneficial for consistent production. However, it also creates inherent vulnerabilities, such as limited genetic diversity and the progressive accumulation of pathogens. Genetic homogeneity implies that the emergence of a novel or aggressive pathogen can lead to rapid spread across an entire crop population. Thus, the consequences are often severe, manifesting as substantial yield losses and systemic economic instability [1,2]. In addition, over successive planting cycles, particularly in the absence of clean planting material, pathogens and opportunistic microorganisms accumulate within the host tissues. This buildup precipitates a phenomenon known as “seed degeneration” [3]. It also increases the probability of mixed infection, which frequently results in complex disease syndromes that are difficult to diagnose and manage [4,5,6,7].

The interaction between multiple pathogens within a single host can trigger synergistic effects that exacerbate disease severity. For example, coinfections with diverse viral species can enhance individual viral pathogenicity, thereby complicating disease management strategies [8,9,10]. In *Musa* species (bananas and plantains), coinfection with multiple viruses, such as Banana streak virus (BSV) and Cucumber mosaic virus (CMV), is relatively common. For example, a study based in Malawi reported that 9% of banana samples exhibited multiple viral infections [11]. The presence of BSV alongside other viruses complicates disease management approaches, as mixed infections can amplify symptom expression and exacerbate the overall impact on banana plants [12]. Recent advances in high-throughput sequencing applied to plant virus detection require a broader perspective on mixed infections and their implications for diagnostics and field management [13]. 

### 1.1. Sweet Potato

Mixed viral infections are common in sweet potato and are exacerbated by the rapid multiplication of the crop via vegetative propagation using cuttings. This mode of propagation increases the risk of pathogen accumulation. Sweet potato feathery mottle virus (SPFMV) is among the most prevalent and geographically widespread viruses affecting sweet potato. Its propensity to coinfect with other viruses contributes to devastating disease complexes, most notably sweet potato virus disease (SPVD). SPVD results from a potent synergistic interaction between SPFMV and members of the sweet potato chlorotic stunt virus (SPCSV) group [14]. These coinfections can lead to the degeneration of local landraces and severely impair plant physiological functions, resulting in yield losses of up to 90% [15,16].

### 1.2. Potato

Potato is perhaps the most extensively studied vegetatively propagated crop in terms of seed degeneration and pathogen interactions. Mixed viral infections in potato can produce synergistic or antagonistic outcomes depending on the specific viral combination, host cultivar, tissue type, and the timing of infection. A typical example of synergism involves potato virus X (PVX). PVX coinfections can increase the titer and symptom severity of other major potato viruses, including potato virus Y (PVY), potato virus S (PVS), and potato leafroll virus (PLRV). Coinfections of PVX and PVY produce rugose mosaic disease, characterized by severe necrosis and stunting. Interestingly, PVX also exacerbates diseases caused by fungal and oomycete pathogens, such as *Verticillium dahliae,* while conversely decreasing tuber susceptibility to *Fusarium roseum* and *Phytophthora infestans* [17].

Antagonism in potato occurs primarily through cross-protection and superinfection exclusion. PVY can evolve through the accumulation of mutations and recombination between strains, generating at least nine recombinant patterns of non-recombinant strains ofPVT^O^ and PVY^N^. Among these, the three principal PVY recombinant strains are PVY^NTN^, PVY^N:O^, and PVY^N-Wi^. Interestingly, PVY^NTN^ and PVY^N-Wi^ infections are asymptomatic or induce only mild symptoms in foliage. Scholars have demonstrated that PVY^N-Wi^ can provide cross-protection against a more damaging PVY strain (PVY^NTN^-PVY^NW^) through competitive advantages, highlighting a mechanistic basis for antagonism that may reduce symptom severity under specific conditions [18].

### 1.3. Banana and Plantain

In *Musaceae*, mixed infections are not limited to viruses. In particular, bacterial pathogens (e.g., *Ralstonia solanacearum*, *Xanthomonas campestris* pv. *musacearum*, and *Erwinia carotovora*) and fungal pathogens (e.g., *Fusarium oxysporum* f. sp. *cubense* (Foc)) frequently co-occur. As vascular pathogens, they are disseminated primarily through infected planting material. Overlapping symptoms from multiple pathogens create complex diagnostic scenarios that hinder effective treatment and containment [19].

### 1.4. Cassava

Cassava, a staple crop across Africa, faces persistent threats from systemic infections. The most devastating scenario involves coinfections of cassava brown streak virus (CBSV) and viruses causing cassava mosaic disease (CMD), which result in increased virulence and severe yield impacts [20]. Mixed infections intensify symptom severity due to synergistic interactions and reveal complex resistance dynamics. For example, African cassava mosaic virus (ACMV) DNA-A confers resistance to ACMV and East African cassava mosaic Cameroon virus (EACMCV) in *Nicotiana benthamiana*. This resistance leads to symptom remission and recovery from infection, supporting the concept of cross-protection among these related geminiviruses. Notably, ACMV DNA-A can inhibit EACMCV infection, resulting in delayed symptom development or even complete suppression in coinfected plants [21].

In the Americas, cassava plants affected by cassava frog skin disease often harbor mixed infections with viruses from multiple families: *Secoviridae* (e.g., cassava torrado-like virus), *Alphaflexiviridae* (e.g., cassava new alphaflexivirus), and *Luteoviridae* (e.g., cassava polero-like virus, CsPLV) [4].

### 1.5. Mixed Infections and Seed Degeneration in Vegetatively Propagated Crops

Not all mixed infections result in rapid disease escalation. Mixed infections contribute to seed degeneration, a process in which pests and pathogens accumulate on or within planting material over successive on-farm propagation cycles. Such accumulation degrades seed quality and reduces yield [22]. This is particularly critical in cassava, when the adoption of certified seed and seed replacement rates remain low [23]. Conversely, several mixed infections are reported to exert beneficial impacts. In sugarcane, coinfection with *Bacillus megaterium* and *Bacillus mycoides* has demonstrated nitrogen-fixation and biocontrol activity against *Sporisorium scitamineum* and *Ceratocystis paradoxa*. This is likely linked to the upregulation of free radical metabolism genes, contributing to greater pathogen tolerance [24].

In the absence of periodic seed system refreshment, these mixed infections drive the process of accumulation of pathogens in planting material, markedly compromising agricultural productivity. This review aimed to provide a comprehensive synthesis of the mechanisms governing mixed infections, highlighting pivotal examples of synergism and antagonism interactions across major starch crops. Furthermore, we examine the underlying molecular mechanisms of infection, host physiological and immune responses, and current advancements in diagnostic technologies. Finally, we evaluate integrated management strategies to mitigate the impact of multipathogen systems in an increasingly complex phytosanitary landscape.

## 2. Characteristics of Vascular-Limited Pathogens in Plants

Vascular-limited pathogens represent a distinct class of phytopathogens that exclusively colonize the plant’s circulatory system, namely, the phloem and xylem. This review focuses specifically on these pathogens as their systemic nature allows them to persist and accumulate within clonal lineages of vegetatively propagated crops. Unlike localized foliar pathogens, vascular colonizers bypass external barriers and occupy a specialized niche that facilitates efficient systemic dissemination, rendering them difficult to detect, exclude from planting materials, and manage through traditional breeding [25,26]. The key physiological differences between phloem- and xylem-limited pathogens are summarized in Table 1.

Xylem and phloem constitute the primary vascular tissues in higher plants, responsible for the translocation of water, minerals, and photoassimilates. These tissues are frequently targeted by specialized pathogens, resulting in substantial agricultural losses. Interestingly, although the phloem is metabolically active and enriched with sugars and nutrients, the majority of vascular pathogens preferentially colonize the xylem, which is comparatively nutrient-deficient. This apparent paradox may be attributed to the structural and physiological properties of the respective tissues. The phloem is composed of living cells with high osmotic pressure and active metabolic processes, creating a restrictive environment for passive colonization. In contrast, the xylem comprises nonliving tracheary elements with low osmotic potential and reduced defense activity, rendering it more accessible to opportunistic colonizers. Consequently, successful phloem colonization often requires vector-mediated transmission, typically via phloem-feeding insects or mechanical intervention through practices such as grafting. Phloem-limited pathogens include a range of fastidious organisms such as rickettsias, spiroplasmas, phytoplasmas, and numerous viruses [25].

Pathogens that invade the xylem include a diverse group of bacteria, fungi, and oomycetes, many of which are associated with vascular wilt diseases. These pathogens typically induce symptoms that progress in an acropetal direction, from the base toward the apex of the plant or acropetal (e.g., *Verticillium* wilt or Mal secco of citrus), causing vascular discoloration, chlorosis, foliar yellowing, and stunted growth [26]. Most xylem-invading pathogens are soilborne and gain entry through root wounds or natural openings, such as stomata and hydathodes. Common examples include *Verticillium dahliae, Fusarium oxysporum*, *Ralstonia solanacearum*, *Rhizoctonia solani*, and *Phythium* spp. In contrast, certain pathogens, such as species in the genera *Xanthomonas* and *Clavibacter* and the fungal pathogen *Ceratobasidium theobromae,* are primarily disseminated via wind or rain splashes and may infect aerial parts of the plant [27]. Moreover, vascular wilt pathogens such as *Xylella fastidiosa* bypass surface barriers entirely as they are transmitted directly into the xylem through insect vectors, particularly xylem-feeding leafhoppers [28].

Phloem-invading pathogens are difficult to detect due to their low titers within host tissues and the manifestation of nonspecific variable symptoms that develop gradually over time [25]. While low titer is a common challenge, this characteristic may vary among specific phloem-limited species. These pathogens generally exhibit complex infection cycles involving plant hosts and insect vectors. A substantial obstacle in the study of phloem-limited bacteria is that most remain unculturable in vitro, preventing fulfillment of Koch’s postulates. Consequently, such bacterial species are classified under the provisional taxonomic status “*Candidatus*”.

Phloem-limited bacteria generally possess small, highly reduced genomes and lack many genes essential for core metabolic functions. This genomic reduction reflects an evolutionary adaptation to the nutrient-rich yet highly specialized phloem environment, resulting in dependence on companion cells to facilitate essential metabolic and reproductive processes. These pathogens are broadly categorized into two groups based on the presence or absence of a cell wall. Among phloem-restricted bacteria lacking cell walls are species such as *Candidatus Phytoplasma asteris* and *Spiroplasma kunkelii.* In contrast, phloem-limited bacteria with a cell wall include *Candidatus Liberibacter asiaticus* (CLas), *Candidatus Phlomobacter fragarie*, *Candidatus Arsenophonus phytopathogenicus*, and *Serratia marcensces* [29].

An additional, distinct group of phloem-invading pathogens comprises phloem-limited viruses, such as Citrus tristeza virus, PLRV, and Squash leaf curl virus. These viruses are primarily confined to phloem tissues, where they replicate and are systemically transported, although their movement is generally more restricted than that of non-phloem-limited viruses. Phloem-limited viruses rely on specific insect vectors—particularly phloem-feeding hemipterans such as aphids and whitefly—for transmission. Their dissemination is governed by unique interactions between the insect vector, the virus, and the host plant. For example, certain phloem-limited viruses, such as Beet yellows virus, are transmitted in a semi-persistent manner. Under such cases, viral acquisition and inoculation occur during specific phases of the aphid’s probing behavior, specifically during the early subphases of intracellular stylet penetration into phloem cells [30]. Furthermore, viruses such as Tomato yellow leaf curl virus (TYLCV) exploit endogenous transport systems, including plasmodesmata and phloem vasculature, to facilitate movement from source to sink tissues. This systemic translocation is driven by the bulk flow of phloem sap, which serves as a conduit for the dissemination of viral particles throughout the host. Beyond facilitating movement, the virus–host interaction often induces profound physiological changes. In particular, the vascular system carries nutrients and viral particles that may trigger the formation of neoplastic tissues that serve as sites for viral replication [31].

The mechanisms of infection and systemic movement in plants during fungal and viral diseases are complex, involving intricate interactions between pathogens and their hosts. Fungal pathogens are classified into three broad categories based on their lifestyle and mode of interaction: biotrophic, necrotrophic, and hemibiotrophic. Each category employs distinct infection strategies and host manipulation mechanisms essential for pathogen survival and proliferation [32]. Despite diverse nutrient acquisition strategies, all pathogenic fungi are subject to recognition by the plant’s innate immune system, which consequently triggers specialized defense responses.

The pathogen–host recognition process begins with surface proteins on spores that mediate attachment to plant surfaces. Under favorable environmental conditions, high humidity induces spores to produce the germ tube that subsequently differentiates into an appressorium. This specialized structure facilitates attachment and exerts mechanical pressure to penetrate the host surface via a penetration peg (penetrating hypha), allowing the fungus to initiate colonization via invasive hyphae.

This infection process is highly regulated. For example, in *Aspergillus fumigatus*, specific surface proteins such as CcpA are essential for virulence. These proteins enable the fungus to evade the host immune system by masking cell wall components that would otherwise trigger a defense response. Moreover, the composition of conidial surface proteins varies significantly across fungal species, directly influencing their pathogenic potential. For example, *A. fumigatus* conidia possess hydrophobin proteins that prevent immune recognition of *β*-1,3-glucans, a conserved component of fungal cell walls [33].

Hydrophobins, small amphiphilic proteins produced by fungi, are crucial for the formation and functionality of appressoria. These proteins enhance the hydrophobicity of the appressorium, facilitating adhesion to the plant cuticle and modulating its physical properties. Hydrophobins also alter the surface tension and promote cell aggregation, ensuring the structural integrity of the infection structure. In particular, the interaction between hydrophobins and lipid droplet-coating proteins regulates lipid metabolism and virulence in genera such as *Colletotrichum* [34].

Appressorium formation is often triggered by hydrophobic surfaces that mimic the host’s cuticular environment or by specific chemical signals such as cutin monomers from the plant epidermis. Several signaling pathways regulate the transition from spore germination to appressorium development. Fungi sense various physical and chemical stimuli to aid host penetration, either via mechanical force or through the localized secretion of lytic enzymes. A primary regulator of morphogenetic reorganization is the Ras GTPase signaling pathway, which acts upstream of cyclic AMP (cAMP) and mitogen-activated protein kinase (MAPK) pathways [35].

Beyond hydrophobins, various secreted effectors are essential for invasion and immune suppression. In *Ustilago maydis*, LysM proteins contribute notable to virulence by modulating the host response [36]. The success of colonization is ultimately dictated by time-sensitive interplay between fungal pathogenicity factors, environmental signals, and the host’s physiological state.

Unlike fungi, viruses lack independent metabolic machinery and must hijack host cellular processes for replication. The infection cycle begins with the introduction of viral particles into the plant cell, typically through mechanical wounding or insect vectors. Once inside, the viral genome utilizes host machinery for replication and the assembly of new virions. Systemic infection is achieved through two primary movement mechanisms: (1) cell-to-cell movement, where viral particles or genomes travel via plasmodesmata, often facilitated by virus-encoded movement proteins (MPs) that modify these channels by enlarging the central cavity or forming specialized tubules (e.g., in *Bromoviridae* and *Tospoviridae*) [37]; and (2) long-distance transport, where viruses utilize the host’s vascular system (primarily the phloem) for systemic dissemination, driven by the bulk flow of photoassimilates.

Viral transmission strategies vary markedly across pathogens. Insect vectors such as aphids, thrips, and whiteflies transmit viruses in either a persistent (requiring prolonged feeding) or nonpersistent (brief feeding) manner [38]. In addition, viruses may be maintained across generations through vertical transmission, including seed transmission (e.g., Tomato yellow leaf curl virus) and transovarial transmission, where the virus passes from an infected female vector to her progeny [39]. Viruses can also spread via mechanical means, where viral particles are transferred through physical injury or contact with infected plant tissue—a method primarily used by nonpersistent viruses.

Once the virus enters the plant system, the infection progresses through a series of interlinked phases: virion uncoating, viral RNA translation, assembly of the viral replication complex, and genomic replication. These steps are followed by cell-to-cell movement and eventual systemic translocation [37]. Specific genetic instructions, including replication genes, movements proteins (MPs), and gene silencing suppressors, are vital for success. Any disruption in these components results in restricted movement, lower viral titers, and attenuated symptoms [40].

Virus-encoded MPs can modify plasmodesmata by either enlarging the central cavity or forming specialized tubules, a mechanism characteristic of the *Bromoviridae*, *Caulimoviridae*, *Secoviridae*, and *Tospoviridae* families [37]. For example, Cauliflower mosaic virus utilizes the endocytic pathway and relies on host proteins for tubule formation. In contrast, potyviruses such as Turnip mosaic virus move systemically without tubule formation, instead utilizing viral proteins, including P3N-PIPO, and host factors (e.g., SYTA and RHD3) for intracellular trafficking and vesicle-mediated transport [41].

The systematic spread of viral RNA and protein through the vascular system eventually leads to widespread physiological changes. These interactions can modulate the host’s metabolism and gene expression to favor viral replication, often manifesting as systemic symptoms such as chlorosis, necrosis, and stunted growth, which severely impact plant health and agricultural yield.

Plant viruses may also be disseminated via mechanical transmission, whereby viral particles are transferred through physical injury or contact with infected plant tissues. This mode of spread is generally less significant for persistent viruses, which require a biological vector for successful inoculation. In contrast, vertical transmission through seeds—denoted as seed transmission—enables certain viruses to persist within plant populations across multiple generations. This mechanism is particularly critical for viruses such as TYLCV, which can accumulate within seeds, thereby ensuring the infection of subsequent generations [42].

### Mixed Infection of Meristematic Tissues

In plants propagated via botanical seeds, meristematic tissues are typically devoid of pathogens, a phenomenon that limits pathogen accumulation. However, specific viruses, bacteria, and other fastidious organisms are capable of breaching these protective barriers. Coinfecting meristematic sites can lead to persistent systemic infections, aberrant developmental patterns, and vertical transmission to progeny or vegetatively propagated clones. Many plant viruses, including members of the genera *Tobamovirus* and *Cucumovirus*, induce the expression of host enzymes such as *β*-glucanases and pectin methylesterases, which increase the size exclusion limit of the plasmodesmata (PD). This physiological modification enhances viral trafficking into the meristematic zone. Moreover, certain persistent viruses in the families *Totiviridae* and *Partitiviridae* lack classical MPs; instead, they replicate directly within meristematic cells, thereby bypassing conventional PD-mediated transport requirements. The ability of specific viruses to persist within the meristems without active replication may represent a form of latent tropism, allowing pathogens to evade immune surveillance and elimination during vegetative propagation, only re-emerging when environmental conditions become favorable [43].

The viral suppression of host RNA interference (RNAi) defenses further complicates these interactions, as one virus may compromise meristematic barrier functions, thereby facilitating the invasion by a second pathogen into tissues it would otherwise be unable to reach. For example, Tobacco rattle virus (TRV) encodes a suppressor of RNA silencing that enables colonization of the meristematic tissues of *Nicotiana benthamiana*. During coinfection with Tobacco mosaic virus (TMV), the RNAi-suppressed environment created by TRV permits TMV to access and replicate within the meristem, demonstrating a clear case of pathogen facilitation [44].

Beyond RNAi suppression and enzymatic modification, the success of mixed infections in meristematic tissues is increasingly attributed to hormonal crosstalk and epigenetic reconfiguration. Pathogens may synergistically modulate phytohormone gradients, particularly auxin and salicylic acid, to alter the cellular environment of the apical dome. Furthermore, “pioneer” pathogens can facilitate the entry of secondary viruses by modifying the protophloem unloading zone, thereby overcoming the spatial isolation of the meristem. This suggests that the meristematic barrier is not a static defense but rather a dynamic interface regulated by the cumulative metabolic load of the infecting viral complex [43].

## 3. Mixed Infections: Diagnosis, Occurrence, and Epidemiology

### 3.1. Advanced Molecular Diagnostic Tools

#### 3.1.1. DNA Barcoding and High-Resolution Phylogenetic Markers

DNA barcoding is a high-precision molecular taxonomic tool used to identify and characterize species based on standardized, short genomic regions. The efficacy of barcoding relies on genetic markers that are sufficiently conserved for universal amplification yet polymorphic enough to discriminate closely related species. The integration of next-generation sequencing (NGS), Oxford Nanopore Technologies, and PacBio long-read sequencing has enhanced the barcoding resolution, particularly when analyzing complex metagenomic samples from mixed infections.

In fungi, several genetic loci are employed to resolve taxonomic relationships. Among them, γ-actin (ACT), β-tubulin (TUB2), and translation elongation factor 1-α (TEF1-α) are widely used markers, while the internal transcribed spacer (ITS) region of the ribosomal RNA gene cluster remains the universal barcode. Each haploid fungal genome contains multiple tandemly repeated copies of the ITS region, ensuring reliable amplification from low-concentration samples. The ITS region (approximately 600 bp) consists of two highly variable spacers (ITS1 and ITS2) separated by the conserved 5.8S rRNA gene, providing an optimal balance between universality and species-level resolution [45].

#### 3.1.2. Fluorescent Probes and Sensors

Fluorescent technologies offer rapid, real-time in situ detection with high sensitivity and selectivity, utilizing molecular reporters that emit light upon binding to specific targets or in response to biochemical changes associated with pathogen-induced stress [46]. This diverse diagnostic toolkit includes nanomaterial-enhanced nanoprobes that leverage quantum dots or metal–organic frameworks to detect low-abundance pathogen DNA in complex matrices, as well as molecular beacon probes—sequence-specific nucleic acids that restore fluorescence upon target binding and are frequently applied in real-time PCR. Furthermore, fluorescence in situ hybridization employs fluorescently labeled nucleic acid probes to target pathogen rRNA or DNA directly within plant tissues, enabling the precise visualization and localization of microbial colonization patterns [47].

#### 3.1.3. High-Resolution Melting Analysis

High-resolution melting (HRM) is a cost-effective, closed-tube post-PCR method that discriminates DNA sequence variants based on the melting behavior of amplicons. Differences in melt profiles reflect single-nucleotide polymorphisms or allelic variation [48]. For example, multiplex HRM analysis can identify trichothecene mycotoxin chemotypes produced by *Fusarium graminearum*, the causal agent of Fusarium head blight (FHB) [49].

### 3.2. Integrated Detection Strategies for Mixed Infections

Most mixed infections involve fastidious pathogens that cannot be easily isolated or cultured, preventing the fulfillment of Koch’s postulates. Consequently, identification relies on molecular techniques. Although conventional approaches (e.g., symptom observation and selective media) remain useful, they are often time-consuming and require extensive taxonomic expertise [50].

#### 3.2.1. Immunological, PCR Techniques, and Isothermal Methods

Immunological methods, such as enzyme-linked immunosorbent assays, are widely utilized for antigen–antibody detection but are limited by reagent shelf life and batch variability [50]. In contrast, nucleic acid-based methods, including polymerase chain reaction (PCR) and loop-mediated isothermal amplification (LAMP), offer superior specificity and can be adapted for point-of-care (POC) field diagnostics [51]. To further streamline field detection, electrochemical DNA biosensors—particularly those using gold nanoparticles (AuNPs)—have been developed to enhance signal efficiency by providing a larger surface area for chemical reactions [52].

To enhance high-throughput screening and the simultaneous amplification of multiple pathogens, scholars have developed various modified PCR platforms for rapid nucleic acid detection, including continuous-flow PCR [53], digital PCR [54], droplet PCR [55], insulated isothermal PCR [56] and ultrafast photonic PCR [57]. However, the requirement for precise temperature control on miniaturized thermal cyclers remains a significant technical challenge. Consequently, isothermal amplification methods have emerged as more suitable alternatives for in-field disease detection. Representative techniques include LAMP, nucleic acid sequence-based amplification [58], strand displacement amplification, recombinase polymerase amplification, and helicase-dependent amplification. Among these, LAMP is most extensively adopted for POC applications due to its robustness and simplicity [59].

A primary limitation of these targeted techniques is their reliance on prior knowledge of pathogen sequences, which restricts their utility in detecting emerging diseases or complex mixed infections. This constraint is addressed by integrating NGS techniques, facilitating the simultaneous detection of multiple pathogens from a single sample and the identification of novel or unexpected disease agents.

#### 3.2.2. Next-Generation Sequencing Techniques

Among the recent advancements in NGS, metagenomic next-generation sequencing (mNGS) has emerged as a transformative approach that enables simultaneous identification of multiple pathogens within a single sample without prior sequence knowledge. Metagenomics is instrumental in characterizing the multispecies nature of plant-associated microbiomes, particularly those involved in complex pathogenesis.

Recent studies have demonstrated the efficacy of mNGS in revealing the diversity of potential pathogens associated with various crop diseases, moving beyond the detection of single agents to a more comprehensive analysis of the pathobiome [60,61]. Notably, this untargeted approach allows for the discovery of known and previously unknown or unexpected pathogens that may contribute to crop disease complexes, offering a more holistic understanding of plant health and disease etiology [34].

Nanopore sequencing has emerged as another novel method, distinguished by its real-time analysis and long-read capabilities. These features have proven highly efficient for the detection and characterization of plant viruses, particularly in the identification of yam viruses and other complex pathogens. The inherent portability and rapid turnaround time of nanopore platforms facilitate their application in field-based diagnostics, allowing for real-time response to emerging disease outbreaks. However, while nanopore sequencing offers speed and portability, other long-read platforms, such as the PacBio Sequel (Pacific Biosciences, Menlo Park, CA, USA), currently provide higher raw-read accuracy. Consequently, further technological refinements are required to enhance the reproducibility and standardization of these long-read methods within the field of phytopathology [62].

### 3.3. Occurrence and Epidemiology of Mixed Viral Infections

Tropical crops critical to global food security, including cassava, sweet potato, maize, soybean, and potato, are frequently afflicted by complex mixed viral infections (Table 2). These coinfections are often perpetuated through vegetative propagation, which facilitates the cumulative buildup of viral titers over multiple growing cycles.

#### 3.3.1. Cassava (*Manihot esculenta*)

Cassava is highly susceptible to mixed infections that exacerbate disease severity. Cassava mosaic disease (CMD) is a primary example resulting from synergistic interactions between various *Begomovirus* species, such as ACMV and the Uganda strain of East African cassava mosaic virus (EACMV-UG) [63]. Furthermore, cassava brown streak disease (CBSD) results from coinfection of CBSV and Ugandan cassava brown streak virus. For molecular diagnostic applications, as these viruses often occur as complexes, multiplex PCR and mNGS are essential for distinguishing between closely related species and strains. In addition, nanopore sequencing is increasingly employed for real-time surveillance in cassava-growing regions [64].

#### 3.3.2. Sweet Potato (*Ipomoea batatas*)

The most devastating mixed infection in sweet potato is SPVD, characterized by a synergistic interaction between the crinivirus SPCSV and the potyvirus SPFMV. SPCSV suppresses the host’s RNAi defenses, leading to a significant increase in SPFMV accumulation, stunted growth, and leaf deformation [16]. A similar synergy occurs in sweet potato severe mosaic disease, involving SPCSV and sweet potato mild mottle virus (SPMMV). Reverse transcription-loop-mediated isothermal amplification (RT-LAMP) has been developed for SPVD to provide rapid, field-based detection of the SPCSV “facilitator” virus, which is often present at low titers but critical for disease progression [65].

#### 3.3.3. Maize and Soybean

Maize lethal necrosis is attributed to the synergism between maize chlorotic mottle virus and sugarcane mosaic virus (SCMV). This interaction leads to severe necrosis and a drastic reduction in chlorophyll and ribosomal RNA ratios [66]. Similarly, soybean is affected by complexes involving soybean vein necrosis virus, bean pod mottle virus and soybean mosaic virus. PCR (dPCR) and TaqMan-based real-time PCR are frequently employed to precisely quantify viral loads in these two cereals, allowing scholars to measure the exact degree of synergistic viral multiplication [67].

#### 3.3.4. Potato (*Solanum tuberosum*)

Potato crops exhibit marked synergistic effects during coinfections of potato virus Y (PVY) (*Potyviridae*) and *potato* virus X (PVX) (*Alphaflexiviridae*), resulting in significantly higher yield losses than single infections [68]. Interactions between PVY and potato virus S (PVS) (*Betaflexiviridae*) similarly enhance viral virulence [69]. HRM analysis is particularly effective in potato diagnostics to discriminate between the numerous necrotic and non-necrotic strains of PVY that often co-exist in mixed infections.

### 3.4. Environmental and Host Mediators of Coinfection

The epidemiology of mixed infections is a dynamic phenomenon shaped by environmental and microbial factors. Nutrient availability, particularly nitrogen supply, and the composition of soil microbial communities exert a significant influence on plant susceptibility and the outcome of pathogen competition [70]. Furthermore, the order of pathogen arrival (priority effects) can dictate whether an interaction is synergistic or antagonistic.

Environmental stressors, notably climate change, play an increasing role in plant-disease management. Altered temperature and precipitation patterns influence vector migration and pathogen replication rates, potentially leading to a higher frequency of novel mixed-infection outbreaks [71]. These climate shifts may alter the geographic distribution and virulence of bacterial and fungal pathogens, further complicating disease management strategies [72].

**Table 2:** Overview of disease complexes, associated pathogens, interaction types, and diagnostic approaches across major crops.

Crop/Host	Disease/Complex	Associated Pathogens	Interaction Type	Mechanism & Diagnostic Tools	References
Cassava	CMD & CBSD	ACMV + EACMV-UG; CBSV + UCBSV	Synergy	Vegetative propagation buildup. **Tools:** Multiplex PCR, mNGS, Nanopore sequencing.	[20,63,64]
Cassava	CMD	ACMV DNA-A vs. EACMCV	Antagonism	Remission and recovery via cross-protection.	[21]
Sweet Potato	SPVD & Severe Mosaic	SPCSV + SPFMV; SPCSV + SPMMV	Synergy	SPCSV suppresses host RNAi, increasing SPFMV titers. **Tools:** RT-LAMP.	[14,16,65]
Maize	Maize Lethal Necrosis (MLN)	MCMV + SCMV	Synergy	Drastic reduction in chlorophyll and rRNA. **Tools:** dPCR, TaqMan-based qPCR.	[66]
Soybean	Viral Complex	SVNV, BPMV, and SMV	Synergy	Multi-virus interaction exacerbates loading. **Tools:** TaqMan qPCR.	[67]
Potato	Mosaic/Necrotic Complexes	PVY + PVX; PVY + PVS	Synergy	Significant yield loss and enhanced virulence. **Tools:** HRM analysis for strain discrimination.	[68,69]
Banana	Sigatoka Disease	*Mycosphaerella* spp. (*fijiensis*, *musicola*, *eumusae*)	Synergy	Heightened virulence in foliar tissue when species co-occur.	[73,74]

ACMV: *African cassava mosaic virus*; EACMV-UG: *East African cassava mosaic virus*–Uganda variant; EACMCV: *East African cassava mosaic* Cameroon virus; CBSV: *cassava brown streak virus*; UCBSV: *Ugandan cassava brown streak virus*; SPCSV: *sweet potato chlorotic stunt virus*; SPFMV: *sweet potato feathery mottle virus*; SPMMV: *sweet potato mild mottle virus*; MCMV: *maize chlorotic mottle virus*; SCMV: *sugarcane mosaic virus*; SVNV: *soybean vein necrosis virus*; BPMV: *bean pod mottle virus*; SMV: *soybean mosaic virus*; PVY: *potato virus Y*; PVX: *potato virus X*; and PVS: *potato virus S.*

## 4. Pathogen–Pathogen Interactions in Mixed Infections

Although viral interactions are frequently emphasized in phytopathology, mixed infections in vegetatively propagated crops also involve complex synergistic and antagonistic relationships between fungi, bacteria, and oomycetes. These interactions can fundamentally alter disease dynamics, often bypassing host defenses or modifying the cellular environment to facilitate secondary colonization. A comparative overview of these interactions, including associated pathogens, mechanisms, and diagnostic tools, is provided in Table 2.

### 4.1. Synergistic Pathogen–Pathogen Interactions

Synergy occurs when two or more pathogens cooperate to exacerbate disease severity beyond the cumulative effects of individual infections. This is particularly evident in soilborne disease complexes, such as cassava root rot. This condition is caused by a consortium of fungi and oomycetes, including species within the genera *Phytophthora*, *Fusarium*, *Lasiodiplodia*, *Neoscytalidium*, and *Phytopythium*. Symptom expression is significantly influenced through synergistic interactions between *Fusarium* spp., *Lasiodiplodia theobromae* (formerly *Botryodiplodia theobromae*), and *Armillaria* spp. [73].

Similarly, Sigatoka disease in banana plants, caused by a complex of *Mycosphaerella fijiensis*, *Mycosphaerella musicola*, and *Mycosphaerella eumusae*, exhibits heightened virulence when at least two of these species are in the same foliar tissue. In viticulture, young grapevine decline involves a synergistic fungal guild including *Ilyonectris* spp., *Phaeomoniella chlamydospora*, and *Togninia* spp., accelerating vine deterioration. Beyond mechanical facilitation, fungi such as those in the genus *Teratosphaeria*, which cause Eucalyptus leaf spot, co-occur to trigger more severe symptomatic responses than single-species infections [73,74].

Synergism involves also bacterial pathogens. Tomato pith necrosis is driven by a bacterial complex involving eight species across the genera *Pseudomonas*, *Ewinia*, and *Dickeya.* Specifically, coinfection of *P. corrugata* with either *P. marginalis* or *P. mediterranea* leads to substantial enhanced tissue degradation. Interkingdom synergism also occurs. For example, fusaric acid secreted by *F. oxysporum* suppresses the expression of genes regulating the antimicrobial activity of 2,4-diacetylphloroglucinol (DAPG), thereby predisposing wheat to subsequent *P. fluorescens* infection [75].

### 4.2. Antagonistic Interactions and Pathogen Competition

In contrast, antagonistic interactions occur when one pathogen suppresses the growth, replication, or virulence of another. In South American cassava, cassava common mosaic virus may be attributed to viral antagonism, where one entity interferes with the systemic movement or replication of a competitor [76]. Furthermore, biological control agents such as *Trichoderma aureoviride* and *Trichoderma hamatum* exhibit antagonism against root rot pathogens through nutrient competition and the secretion of inhibitory enzymes [77].

Antagonism is also well-documented in cereal crops. In wheat, the presence of the necrotroph *Zymoseptoria tritici* (Septoria tritici blotch) reduces the colony size and reproductive capacity of the biotroph *Blumeria graminis* f.sp. *tritici* (Bgt) (powdery mildew). Interestingly, this suppression occurs during the latent phase of *Z. tritici*, suggesting a physiological reconfiguration of the host tissue before visible symptoms appear [78].

In maize, interactions between *Ustilago maydis* and *Fusarium verticillioides* reduce biomass in the former due to the toxic secondary metabolites secreted by *F. verticillioides*. In response to this chemical warfare, *U. maydis* upregulates genes involved in siderophore biosynthesis and toxin production to compete for niche space and resources, revealing a sophisticated metabolic struggle within the host environment [79].

## 5. Host Responses to Mixed Infections

### 5.1. Xylem-Specific Immunity and Compartmentalization

Plants deploy a sophisticated suite of constitutive and inducible defenses within the xylem, primarily centered on the strategy of compartmentalization to inhibit pathogen spread. Vertical spread within the vessel lumen is physically restricted by the formation of tyloses—protoplasmic outgrowths of xylem parenchyma—and the accumulation of viscous gels. Horizontal spread into adjacent healthy tissues is mitigated by the vascular coating of vessel walls with hydrophobic lignin and suberin.

Simultaneously, xylem infection triggers profound metabolic shifts in adjacent parenchyma cells. This leads to the secretion of a potent chemical cocktail into the xylem sap, comprising pathogenesis-related (PR) proteins (PR-1 through PR-5), enzymatic modifiers such as peroxidases and xyloglucan-specific endoglucanase inhibitor proteins, and diverse secondary metabolites including phenols and phytoalexins [26,80].

### 5.2. Phloem-Based Immunity and Signaling

The phloem constitutes a specialized ecological niche for diverse pathogens, where defense is primarily initiated in the leaf mesophyll via a sophisticated dual-layered immune system. This defense architecture begins with pattern-triggered immunity (PTI), which is activated when surface-localized pattern recognition receptors detect conserved microbe or pathogen-associated molecular patterns (MAMPs/PAMPs), such as the GroEL protein from *Buchnera aphidicola* or flagellin (FlaLas) from *Candidatus Liberibacter asiaticus* [81].

To overcome PTI, pathogens deploy virulence effectors, which plants counteract through effector-triggered immunity (ETI). This secondary layer is mediated by intracellular resistance (R) proteins, typically nucleotide-binding leucine-rich repeat proteins that recognize specific effectors and trigger a robust immune response. However, although ETI is highly effective against mesophyll-invading pathogens, its activation has been notably less documented in response to strictly phloem-inhabiting pathogens, highlighting a potential gap in the immune surveillance of the plant’s vascular network [81].

### 5.3. Defense Dynamics in Mixed Infections

In the context of mixed infections, host immunity can be modulated through two divergent mechanisms: pathogen-triggered host susceptibility and pathogen-triggered host immunity. In the former scenario, a primary pathogen may compromise host defenses to facilitate secondary colonization. For example, fusaric acid secreted by *F. oxysporum* suppresses the expression of genes regulating antimicrobial activity, thereby predisposing the host to *Pseudomonas fluorescens* infection [75]. Conversely, an initial infection can serve as a “vaccine” by priming the plant’s defensive capacity against subsequent attacks, a phenomenon exemplified by non-pathogenic strains of *F. oxysporum* that induce systemic resistance against their pathogenic counterparts in tomato plants [82].

### 5.4. Phytohormone Crosstalk and the Hypersensitive Response

Mixed infections frequently trigger the hypersensitive response (HR), a localized form of programmed cell death characterized by a MAPK signaling cascade, an influx of calcium ions, and a rapid burst of reactive oxygen species [83]. This defensive process is orchestrated by intricate phytohormone crosstalk, primarily involving salicylic acid (SA), which mediates resistance against biotrophic pathogens and induces systemic acquired resistance, and the jasmonic acid (JA) and ethylene (ET) pathways, which typically regulate defenses against necrotrophic pathogens and herbivores [84,85]. The outcome of these coinfections is determined by a metabolic “tug-of-war” between these pathways. Although SA and JA often function antagonistically, their simultaneous activation during mixed infections can lead to a prioritized defense strategy. In this scenario, the plant reconfigures its transcriptional profile and redirects metabolic investments based on the perceived virulence and threat level of the primary pathogen [86].

In summary, plants defend their vascular systems against mixed infections through tissue-specific mechanisms and complex signaling crosstalk. In the xylem, defense relies on compartmentalization, utilizing physical barriers such as tyloses and gels to block vertical spread, and lignin and suberin coatings to prevent horizontal movement alongside the secretion of antimicrobial proteins and secondary metabolites into the sap. In contrast, phloem immunity is driven by a dual-layered system of PTI and ETI, although ETI is less documented in strictly phloem-limited pathogens.

During mixed infections, these defenses are dynamically modulated. A primary pathogen may induce susceptibility by suppressing antimicrobial genes or, conversely, by acting as a “vaccine” by priming the plant’s immune capacity against subsequent attacks. These responses culminate in a sophisticated regulatory balance orchestrated by phytohormone crosstalk, where the SA and JA pathways compete or cooperate to prioritize metabolic investments and trigger the HR based on the most significant pathogenic threat.

## 6. Impacts on Plant Health and Productivity

The influence of mixed infections on plant development is often profoundly damaging, primarily driven by competitive or synergistic dynamics between coinfecting pathogens. When multiple pathogens compete for identical host resources, the resulting physiological stress typically culminates in stunted growth and diminished vitality. Interestingly, interstrain competition—such as that between different strains of the same phytoplasma—may reduce disease severity by partitioning the energy required for their respective life cycle, a phenomenon that can fortuitously benefit the host [87]. Conversely, many mixed infections manifest as synergistic interactions, where the presence of one pathogen enhances the virulence or pathogenicity of another. This dynamic is particularly evident in vegetatively propagated crops, such as cassava afflicted by various *Begomovirus* species (CMD) and sweet potato affected by complexes of *Crinivirus*, *Potyvitus*, and *Ipomovirus* [9].

### 6.1. Yield Implications in Tropical Staples

The yield gap in tropical staples remains a critical challenge for global food security. Although the theoretical maximum yield for cassava under optimal conditions can reach 90 tons of fresh roots (approximately 30 tons of dry matter) per hectare, actual yields in tropical regions remain significantly lower. In 2017, the worldwide average yield was recorded at only 11.08 tons per hectare, with leading producers such as Nigeria achieving an average of 8.75 tons per hectare. Single infections account for substantial losses; however, mixed infections are estimated to increase yield loss incidence by approximately 10% [88].

In sweet potato, the synergistic effects of mixed infections (e.g., SPCSV combined with CMV, SPMMV, or C-6) are even more pronounced. Research indicates that while a single infection may not significantly impact the total storage roots, mixed infections can trigger fresh foliage and storage root reductions within the ranges of 40.2–50.6% and 17.7–23.8%, respectively [89].

### 6.2. Challenges in Management and Disease Dynamics

Managing mixed infections presents a significant challenge as pathogen–pathogen interactions can fundamentally reshape host defense responses and pathogen evolution. These interactions may facilitate horizontal gene transfer, potentially enhancing virulence or allowing pathogens to colonize new ecological niches. Furthermore, the outcome of coinfection, whether synergistic or antagonistic, is often dictated by priority effects and environmental variables. Human-made conditions and climate shifts can further alter these dynamics, potentially favoring the invasion of more aggressive strains [90].

### 6.3. Future Diagnostics and Further Research

A primary longitudinal challenge is identifying the epidemiological traits and metabolic reconfigurations required for a pathogen to optimize nutrient acquisition within novel niches. To unravel these mechanisms, high-throughput multispecies transcriptomics and metabolomics are essential, as they provide a holistic view of the “pathobiome”. However, diagnostic bottlenecks remain. While NGS and metagenomics can identify the total microbial community within samples, these tools often fail to demonstrate the pathogenicity of specific constituents or identify which microbe is the primary driver of the disease [73]. Effective long-term crop management will therefore require an integrated approach that combines robust NGS diagnostics with functional assays to determine the relative contribution of each pathogen within a complex.

## 7. Management Strategies for Mixed Infections on Crops

The effective management of mixed infections begins with an accurate diagnosis, which serves as the foundational step for all subsequent interventions. Precise identification of the pathogens involved—utilizing molecular assay, serological tests, or standardized symptom-based observations—is essential for selecting appropriate control measures. As mixed infections can synergistically modify disease expression and severity, misdiagnosis often results in ineffective or counterproductive management decisions. For example, in cassava systems plagued by severe complexes of *Begomovirus* (CMD) and *Ipomovirus* (CBSD), management strategies are categorized into three pillars: recognition and monitoring, prevention, and therapeutic control [91].

### 7.1. Recognition and Pathogen Monitoring

Recognition of disease occurrence is a prerequisite for its control. However, the ease of detection varies between pathogens. For economically devastating diseases such as CMD and CBSD in Africa, large-scale surveillance programs are required to monitor incidence shifts across geographic and temporal scales. While CMD can be effectively monitored via visual symptoms and standardized survey protocols [92], the cryptic nature of CBSD symptoms requires reinforcement through molecular diagnostic tools to ensure accurate detection [91].

### 7.2. Preventive Measures and Phytosanitation

Prevention is often the most cost-effective management strategy, primarily involving phytosanitation and cultural control. This process begins with ensuring the health of planting material and selecting sites isolated from inoculum sources or unfavorable for insect vectors. In cassava, which is rarely grown in protected environments, the hardening of tissue-cultured plantlets during germplasm exchange represents a rare exception to field-based cultivation. Furthermore, infection can be mitigated by deploying varieties resistant to vector-borne transmission or by managing the vectors themselves—although vector control is infrequently practiced in subsistence cassava framing [91].

### 7.3. Control Interventions and Therapeutic Methods

Once a virus breaches preventive barriers, control interventions must be initiated to inhibit virus replication, movement, or further spread. This includes roguing, namely, the removal of symptomatic or virus-positive asymptomatic plants, as well as sophisticated laboratory techniques. For example, combining thermotherapy (35 °C for one month) with 0.3 mm meristem tip culture can effectively eliminate viral particles, allowing for the propagation of clean seed through tissue culture [93]. The efficacy of phytosanitation depends on the pathogen’s nature. For example, CMD is efficiently transmitted by whitefly, making field isolates difficult to control, while the cryptic symptoms of CBSD make phytosanitation a high-benefit intervention, as cutting-borne infection in CBSD often exceeds vector-mediated spread [94].

### 7.4. Fungicidal Management of Mixed Infections

For cases involving fungal coinfections, fungicides remain a primary management tool and are generally classified into two functional categories based on their mobility and mode of action. Systemic fungicides, such as benomyl, propiconazole, and metalaxyl, are absorbed by the plant and translocated throughout its vascular system, providing comprehensive protection even to tissues not directly reached during application. This systemic mobility is particularly effective against pathogens that do not rely on spores as their primary infective structure, such as *Rhizoctonia* [95]. In contrast, contact fungicides, including chlorothalonil and mancozeb, act primarily on the plant surface to create a protective barrier that prevents fungal spores from germinating or penetrating host tissue. As these chemicals do not move within the plant, they require thorough surface coverage and are most effective when applied preventively before the onset of infection [96].

### 7.5. Breeding for Durable Resistance

The most sustainable approach for managing mixed infections lies in the deployment of resistant genotypes, which utilize conventional resistance mechanisms to restrict viral replication and movement to subeconomic thresholds. In cassava, this resistance is primarily associated with key genetic loci: the polygenic, recessive CMD1, derived from *Manihot glaziovii* [97], and the major dominant genes CMD2 and CMD3, identified in Nigerian landraces [98].

Historical breeding efforts have successfully utilized these sources. For example, clones such as 5318/34 were selected for CMD resistance as early as 1958. Subsequent breeding objectives focused on incorporating this resistance into well-adapted local cultivars through crosses involving clone 58308, local varieties, and M. glaziovii. These efforts led to the 1977 release of improved families such as TMS 30395, TMS 63397, TMS 30555, TMS 4(2)1425, and TMS 30572 [99], with varieties including TMS 30359 and TMS 30001 demonstrating near-immunity to CMD. By combining CMD1 and CMD2, breeders have developed high-yielding varieties with durable resistance capable of withstanding the synergistic pressures of mixed infections [100]. For diseases such as CBSD, where natural resistance is limited, RNAi targeting viral coat proteins offers a promising biotechnological alternative [101].

Similar complexities are observed in other vegetatively propagated crops, such as bananas, which face the mixed infections of *Mycosphaerella fijiensis* and *M. musicola*. In these systems, resistance is governed by intricate allelic interactions, including the recessive allele *bs*1 and two additive independent alleles, *bsr*2 and *bsr*3. Intralocus interactions at the *bs*1 locus are associated with the onset of early symptoms, while interactions at the *bsr* loci influence disease progression [102]. Sustainable management relies on quantitative resistance sourced from wild *Musa* species, such as *Musa acuminata* and the diploid Calcutta 4.

Evaluating resistance in these genotypes requires rigorous phenotyping in field or greenhouse settings, utilizing standardized inoculation methods and precise inoculum concentrations. As fungal strains vary in pathogenicity, prior characterization of isolates is essential for reliable results [103]. To accelerate these time-consuming breeding cycles, modern programs increasingly employ molecular markers, such as simple sequence repeats and quantitative trait loci mapping, to facilitate the selection of resistant populations [104,105].

## 8. Future Directions and Research Gaps

A primary concern regarding mixed infections is the emergence of new disease complexes and the inherent difficulty in rapidly identifying their causal agents. The challenge lies not only in detecting the associated microorganisms; however, in distinguishing true pathogens from commensal or transient microbiota. Although NGS and metagenomics have revolutionized diagnostics by generating comprehensive datasets of microbial communities, the presence of a microorganism within a host does not inherently confirm its pathogenicity. Although comparative metagenomic analyses between symptomatic and asymptomatic tissues can isolate potential candidates, this correlative approach often lacks the definitive evidence required for causation.

### 8.1. Re-Evaluating Koch’s Postulates in the Genomic Era

Determining the primary driver of disease within a polymicrobial context requires a critical re-evaluation of Koch’s postulates, which face significant operational limitations in complex pathosystems. Criterion I, the requirement for isolation in pure culture, is technically impossible for obligate pathogens, including phytoplasmas, spiroplasmas, and certain fungi such as *Puccinia* and *Podosphaera.* Furthermore, successful symptom induction under Criterion II is highly sensitive to environmental variables—including temperature, humidity, and host age—and the utilization of specific infective structures. Criteria III and IV, concerning re-isolation and re-infection, are often invalid for synergistic diseases, where a single pathogen may not manifest symptoms in the absence of its coinfectant. This framework is further complicated by quiescent microorganisms. For example, *Xylella fastidiosa* can colonize grapevine xylem vessels as an asymptomatic endophyte for extended periods [106], and Anthracnose pathogens often establish infections months before visible symptoms emerge [107]. Consequently, traditional postulates must be integrated with molecular evidence to accurately define pathogenicity in coinfection scenarios.

### 8.2. Ecological Shifts and Niche Expansion

Research into pathogen–pathogen interactions as a basis for holistic disease management remains underdeveloped. This is a critical gap, as long-term shifts in anthropogenic factors (e.g., the introduction of varieties with major gene resistances) and environmental conditions (e.g., climate change) fundamentally alter pathogen coexistence. Changes in these variables may favor specific pathogens, leading to large-scale invasions or the colonization of novel niches. A notable example is *Ceratobasidium theobromae,* a known cacao pathogen that has recently expanded its host range to include cassava [108]. This highlights the urgent requirement to integrate climate change models with phytopathology to predict emerging single pathogen outbreaks and novel synergistic complexes [109].

### 8.3. Integrative Diagnostic and Management Frameworks

The future of plant pathology lies in the convergence of high-throughput techniques and traditional methodologies. Integrating NGS-based techniques such as multispecies transcriptomics and metabolomics with classical phytopathological assays and environmental modeling can provide a more detailed understanding of pathogen–pathogen–host interactions. Such a holistic framework is essential for the development of integrated control measures capable of mitigating the risks posed by complex, multipathogen diseases in a changing global climate.

## 9. Conclusions

The best-characterized examples of mixed virus interactions include the PVY/PVX-HC-Pro and SPCSV/SPFMV RNase3 systems, highlighting the role of the plant immune system as a central hub for these pathogens to modify and generate synergistic effects.

The biology and molecular interactions of these intermicrobial processes may be important in defining new targets and strategies for disease control. Through molecular techniques, it is possible to identify key host responses for breeding resistance varieties, enabling marker-assisted selection and the timely use of resistance inducers to stimulate the production of phytohormones and prevent fungal infections.

Plant-associated pathogenic microorganisms should be studied with the perspective that antagonistic, mutualistic, or synergistic interactions may be occurring. In most cases, microorganisms detected by NGS or metagenomics are not involved in the disease process, creating challenges for routine diagnostics, such as in surveillance or seed certification, potentially facilitating disease spread.

The correct identification of a disease’s causal agent guarantees early disease diagnosis and informs appropriate disease management strategies. Infection mechanisms vary depending on the microorganism involved. For fungi, a critical step in infection is attachment and host recognition, since the host’s defense responses can prevent successful spore germination or appressorium formation. However, some fungi do not produce spores, or the spores are not the infective structure. Understanding the climatic conditions that favor this process is also of essential importance for developing effective strategies to manage mixed infections. Some fungi only produce spores under high humidity conditions, requiring free water on the leaf surface to germinate. This represents the optimal moment to apply disease management strategies.

It is essential to conduct further research to examine the ecological and molecular dynamics of pathogen interactions, host responses, and environmental influences. Further, the development of integrated disease models and management practices informed by real-world, multipathogen scenarios is equally important. Collaboration among plant pathologists, microbiologists, agronomists, and bioinformaticians is key to advancing our ability to predict, detect, and control complex disease systems in an increasingly interconnected and climate-variable world.

## Figures and Tables

**Table 1:** Comparative features of phloem-limited and xylem-limited pathogens in plant vascular systems.

Feature	Phloem-Limited Pathogens	Xylem-Limited Pathogens
Tissue State	Living cells; high osmotic pressure	Non-living tracheary elements; low defense activity
Nutrient Status	Rich (sugars, photoassimilates)	Nutrient-deficient (water, minerals)
Entry Mechanism	Insect vectors (aphids, whiteflies) or grafting	Soilborne entry via root wounds or insect vectors
Example Pathogens	Phytoplasmas, TYLCV, Citrus tristeza virus	*Fusarium*, *Ralstonia*, *Xylella fastidiosa*
Movement	Systemic (Source-to-sink bulk flow)	Acropetal (Base-to-apex flow)

TYLCV: *Tomato yellow leaf curl virus*.

## Data Availability

No new data were created or analyzed in this study. Data sharing is not applicable to this article.

## Abbreviations

The following abbreviations are used in this manuscript:

ACMV	*African cassava mosaic virus*
BPMV	*Bean pod mottle virus*
BSV	*Banana streak virus*
BYV	*Beet yellows virus*
CaMV	*Cauliflower mosaic virus*
CBSD	Cassava brown streak disease
CBSV	*Cassava brown streak virus*
CMD	Cassava mosaic disease
CMV	*Cucumber mosaic virus*
CsCMV	*Cassava common mosaic virus*
CTV	*Citrus tristeza virus*
EACMCV	*East African cassava mosaic* Cameroon virus
ELISA	Enzyme-linked immunosorbent assay
ETI	Effector-triggered immunity
FISH	Fluorescence in situ hybridization
HDA	Helicase-dependent amplification
HR	Hypersensitive response
HRM	High-resolution melting
HTS	High-throughput sequencing
ITS	Internal transcribed spacer
JA	Jasmonic acid
LAMP	Loop-mediated isothermal amplification
MAPK	Mitogen-activated protein kinase
MCMV	*Maize chlorotic mottle virus*
MLN	Maize lethal necrosis
MP	Movement protein
NASBA	Nucleic acid sequence-based amplification
NGS	Next-generation sequencing
NLR	Nucleotide-binding leucine-rich repeat
ONT	Oxford Nanopore Technologies
PCR	Polymerase chain reaction
PLRV	*Potato leafroll virus*
POC	Point-of-care
PR	Pathogenesis-related
PRR	Pattern recognition receptor
PTI	Pattern-triggered immunity
QTL	Quantitative trait loci
ROS	Reactive oxygen species
RPA	Recombinase polymerase amplification
SA	Salicylic acid
SAR	Systemic acquired resistance
SDA	Strand displacement amplification
SEL	Size exclusion limit
SLCV	*Squash leaf curl virus*
SMV	*Soybean mosaic virus*
SNP	Single-nucleotide polymorphism
SPCSV	*Sweet potato chlorotic stunt virus*
SPFMV	*Sweet potato feathery mottle virus*
SPMMV	*Sweet potato mild mottle virus*
SPVD	Sweet potato virus disease
SSR	Simple sequence repeat
SVNV	*Soybean vein necrosis virus*
TMV	*Tobacco mosaic virus*
TRV	*Tobacco rattle virus*
TuMV	*Turnip mosaic virus*
TYLCV	*Tomato yellow leaf curl virus*
UCBSV	*Ugandan cassava brown streak virus*
VRC	Viral replication complex
XEGIP	Xyloglucan-specific endoglucanase inhibitor protein
